# Supplementary material for: Gel-Based Proteomic Identification of Suprabasin as a Potential New Candidate Biomarker in Endometrial Cancer
Source: Int J Mol Sci. 2022 Feb 14;23(4):2076. doi: 10.3390/ijms23042076 (PMC8880426; doi:10.3390/ijms23042076)
Supplement: Supplementary file 1 [file ijms-23-02076-s001.zip › SUPLEMENTAL TABLE BU.pdf]

Supplementary Table S1: Clinico-pathological characteristics of the 43 EC women enrolled in the study.

**Sample type**	**Age**	**Histotype**	**Grade**	**Stage**	**Biological sources**	**Methodological used**
Tumor	81	Endometroid adenocarcinoma	G2	IB	serum	2D-DIGE and western-blotting
Tumor	76	Endometroid adenocarcinoma	G1	IA	serum	2D-DIGE and western blotting
Tumor	73	Endometroid adenocarcinoma	G1	IB	serum	2D-DIGE and western blotting
Tumor	72	Endometroid adenocarcinoma	G2	IB	serum	2D-DIGE and western blotting
Tumor	82	Endometroid adenocarcinoma	G2	IB	serum	2D-DIGE and western blotting
Tumor	82	Endometroid adenocarcinoma	G2	IB	serum	2D-DIGE and western blotting
Tumor	74	Endometroid adenocarcinoma	G2	IA	serum	2D-DIGE and western blotting
Tumor	67	Endometroid adenocarcinoma	G1	IB	serum	2D-DIGE and western blotting
Tumor	60	Endometroid adenocarcinoma	G1	IA	serum	2D-DIGE and western blotting
Tumor	80	Endometroid adenocarcinoma	G1	IB	serum	2D-DIGE and western blotting
Tumor	51	Endometroid adenocarcinoma	G2	IIIC1	serum	Western-blotting
Tumor	82	Papillary serous carcinoma endometrium	G3	IA	serum	Western-blotting
Tumor	69	Endometroid adenocarcinoma	G3	IIIC1	serum	Western-blotting
Tumor	52	Endometroid adenocarcinoma	G2	IIIC1	Tissue+serum	Western-blotting
Tumor	77	Endometroid adenocarcinoma	G2	II	Tissue+serum	Western-blotting
Tumor	70	Endometroid adenocarcinoma	G2	IA	Tissue+serum	Western-blotting
Tumor	64	Endometroid adenocarcinoma	G3	II	Tissue+serum	Western-blotting
Tumor	53	Endometroid adenocarcinoma	G1	IA	Tissue+serum	Western-blotting
Tumor	58	Endometroid adenocarcinoma	G3	IIIC1	Tissue+serum	Western-blotting
Tumor	70	Endometroid adenocarcinoma	G2	IA	Tissue+serum	Western-blotting
Tumor	79	Endometroid	G2	IB	Tissue+serum	Western-

Supplementary Table S1: Clinico-pathological characteristics of the 43 EC women enrolled in the study.

		adenocarcinoma				blotting
Tumor	68	Endometroid adenocarcinom	G3	IA	Tissue+serum	Western-blotting
Tumor	55	Endometroid adenocarcinoma	G1	IA	Tissue+serum	Western-blotting
Tumor	74	Endometroid adenocarcinoma	G3	IA	Tissue+serum	Western-blotting
Tumor	62	Endometroid adenocarcinoma	G1	IA	Tissue+serum	Western-blotting
Tumor	87	Endometroid adenocarcinoma	G2	II	Tissue+serum	Western-blotting
Tumor	79	Endometroid adenocarcinoma	G3	II	Tissue+serum	Western-blotting
Tumor	77	Endometroid adenocarcinoma	G2	IB	Tissue+serum	Western-blotting
Tumor	60	Serous carcinoma endometrium	G1	IA	Tissue+serum	Western-blotting
Tumor	80	Endometroid adenocarcinoma	G1	IB	Tissue+serum	Western-blotting
Tumor	73	Endometroid adenocarcinoma	G3	II	Tissue	Western-blotting
Tumor	61	Endometroid adenocarcinoma	G2	IA	Tissue	Western-blotting
Tumor	58	Endometroid adenocarcinoma	G3	IB	Tissue	Western-blotting
Tumor	82	Endometroid adenocarcinoma	G2	II	Tissue	Western-blotting
Tumor	64	Endometroid adenocarcinoma	G2	IB	Tissue	Western-blotting
Tumor	57	Endometroid adenocarcinoma	G3	IA	Tissue	Western-blotting
Tumor	54	Endometroid adenocarcinoma	G3	IB	Tissue	Western-blotting
Tumor	75	Endometroid adenocarcinoma	G2	IB	Tissue	Western-blotting
Tumor	72	Endometroid adenocarcinoma	G1	IB	Tissue	Western-blotting
Tumor	76	Endometroid adenocarcinoma	G2	IIIA	Tissue	Western-blotting
Tumor	57	Endometroid adenocarcinoma	G1	IIIC1	Tissue	Western-blotting
Tumor	74	Papillary serous carcinoma endometrium	G3	IB	Tissue	Western-blotting
Tumor	70	Endometroid adenocarcinoma	G2	IA	Tissue	Western-blotting
